# Efficacy and Safety Assessment of a Dietary Supplement in a Rat Model of Osteoarthritis and Dogs with Arthritic Signs

**DOI:** 10.3390/ani15131825

**Published:** 2025-06-20

**Authors:** Geon A Kim, Mi-Jin Lee, Eun Pyo Kim, Gun Ho Heo, Seung Gyu Oh, Se Chang Park, Byeong Chun Lee, Sang O Park

**Affiliations:** 1 Department of Biomedical Laboratory Science, College of Health Science, Eulji University, Uijeongbu 11759, Republic of Korea; kimgeona2020@gmail.com; 2 Department of Veterinary Nursing, College of Health Sciences, Wonkwang University, Iksan 54651, Republic of Korea; gvetdog@daum.net; 3 Naeun Animal Hospital, Uijeongbu 11718, Republic of Korea; kepjjang@hanmail.net; 4 Yedam Animal Hospital, Daejeon 35207, Republic of Korea; yedamamc@gmail.com (G.H.H.); yedamamc@naver.com (S.G.O.); 5 Laboratory of Aquatic Biomedicine, College of Veterinary Medicine, Research Institute for Veterinary Science, Seoul National University, Seoul 08826, Republic of Korea; parksec@snu.ac.kr; 6 Faculty Startup Company SHA Bio Co., Ltd., College of Veterinary Medicine, Seoul National University, Seoul 08826, Republic of Korea; 7 Mammi Dr. Research Institute, Seongnam 13524, Republic of Korea; 8 EreBon Co., Ltd., Icheon 17407, Republic of Korea

**Keywords:** osteoarthritis, BYVET JOINT HEAL, mucopolysaccharide, chondroitin sulfate, undenatured collagen type II, omega 3 fatty acids

## Abstract

BYVET JOINT HEAL^TM^ (BJH) contains mucopolysaccharide protein, chondroitin sulfate, type II collagen, and omega-3 fatty acids, which protect and prevent osteoarthritis (OA)-associated tissue damage and degradation in dogs and cats. This study aimed to generate a novel dietary supplement and evaluate its prevention and therapeutic efficacy in an OA Sprague Dawley rat model induced using monosodium iodoacetate (MIA). Negative control, MIA-induced OA control (MIA), OA rats treated with BJH three weeks after (M+BJH3) and those treated two weeks before and three weeks after OA induction (BJH2+M+BJH3) groups were assigned. M+BJH3 and BJH2+M+BJH3 had similar mean body weight increases until 29 days. BJH2+M+BJH3 showed a significantly higher body weight than M+BJH3 and MIA on the final day. Interleukin-1β in BJH2+M+BJH3 was significantly lower than that in MIA. Tumor necrosis factor-α, aggrecan, matrix metalloproteinases13, and cyclooxygenase-2 levels in M+BJH3 and BJH2+M+BJH3 significantly differed compared to those in MIA. BJH administration before OA induction significantly decreased OA severity and functional recovery. Consuming a BJH supplement showed modifying and chondroprotective effects and significantly reduced cartilage degeneration and inflammation with no side effects. Hence, our findings demonstrate the potential of using BJH as a safe therapeutic and preventive supplement for OA and associated cartilage abnormalities. Also, 30 dogs diagnosed with OA by a veterinarian participated in the clinical trial, and BJH was provided for 8 weeks. Blood tests (CBC, serum chemistry) and joint assessment were performed before and after the feeding, and the effects of a BJH supplement were compared. BJH supplement was easy to administer, and no side effects were reported. Feeding BJH supplementation alone to dogs with arthritis had an overall positive effect on arthritis scores for 8 weeks without any other treatment, including non-steroidal drugs.

## Simple Summary

A dietary supplement containing mucopolysaccharide, chondroitin sulfate, type II collagen, and omega-3 fatty acid was orally administered to a rodent model and dogs with osteoarthritis over five weeks to evaluate their preventive and therapeutic efficacy. The group administered before the induction of osteoarthritis showed a preventive effect compared to the control group in which osteoarthritis was induced in terms of histological results and gene expression related to joint inflammation and damage. In addition, even when administered after the induction of osteoarthritis, there was an improvement effect in cytokines, gene expression, and tissue examination in the joint space. No detrimental effects on kidney and liver function were observed. In dogs with arthritic signs, all clinical evaluation was improved. This confirmed the efficacy and safety of this dietary supplement in osteoarthritis-induced rats and dogs.

## 1. Introduction

Articular cartilage is highly specialized and consists of uniquely designed avascular, aneural, and lymphatic materials synthesized by the sparse chondrocytes. The extracellular matrix is extensively composed of type II collagen and proteoglycans that are responsible for the functional properties of the cartilage. It is an important part of the skeletal muscle disease osteoarthritis (OA), a chronic degenerative bone and joint disease that is a serious concern in companion animals. Although it has long been considered only a cartilage disease, OA has now been widely recognized to affect all joint tissues through inflammatory and degenerative processes [1]. OA is characterized by progressive cartilage degeneration, varying synovitis degrees, collagen network rupture, proteoglycan depletion, and increased collagen and proteoglycan synthesis. Collagen is degraded by matrix metalloproteinases (MMPs), proteolytic enzymes involved in the breakdown of the cartilage extracellular matrix. Many of these proteinases are released under the influence of proinflammatory cytokines such as interleukin-1 (IL-1) and tumor necrosis factor-α (TNF-α), which can enhance prostaglandin E biosynthesis [2]. These signals can exert pain in the articular cartilage and cause lameness in companion animals.

The prevalence of OA in companion animals varies widely; in cats, it ranges from 16 to 91% [3,4,5,6,7,8] and up to 20% in dogs that are more than a year old [9], depending on the studied population. In addition to the impact on the welfare of companion animals, treatment plans are considerably expensive for owners. In particular, the emotional cost of caring for animals suffering from the chronic pain of OA can also cause psychological stress and depression [10]. Current medical treatments for companion animals with OA rely mostly on relieving pain and controlling inflammation such that companion animals can perform daily activities using their joints [11]. However, non-steroidal anti-inflammatory drugs have several adverse effects, such as gastrointestinal hemorrhage and impairing liver and kidney function [12]; their safety has not been confirmed for all ages of companion animals, including dogs [13,14]. Dietary supplements containing some nutrients are increasingly being used for OA management. Therefore, veterinarians recommend consuming ingredients, including chondroitin sulfate and collagen type II, that are naturally synthesized in the body to treat OA, even though this information is not supported by sufficient scientific evidence [15].

Mucopolysaccharide proteins, also known as glycosaminoglycans (GAGs), are unbranched long polysaccharides with a disaccharide structure [16]. One of two main groups of GAGs is sulfated GAGs, chondroitin sulfate. Chondroitin sulfate is primarily sourced from diverse byproducts derived from land and sea animals, including fish bones and fins [17], bovine trachea [18], and chicken keel cartilage [19]. Collagen contributes to approximately 60% of the dry weight of hyaline cartilage and is the main protein in the cartilage extracellular matrix [20]. Collagen type II is cartilage-specific. Therefore, ingesting the precursors that make up these joints as nutrients can protect the joints even when an existing pathologic change has occurred.

Collagen type II can be derived from collagenous tissue, such as bones and cartilage of animals, such as chicken and fish. Undenatured collagen type II is nontoxic for human consumption and is generally recognized as a safe food ingredient [21]. Undenatured collagen type II has been known to exert articular health via oral tolerance. When consumed, type II collagen is believed to be taken up by the Peyer’s patches, the lymphoid tissue neighboring the small intestine. It then activates immune cells that secrete anti-inflammatory cytokines [22,23]. Hence, it can help reduce joint inflammation. A study reported that the effect of an agent containing a mucopolysaccharide protein in a rabbit model caused anterior cruciate ligament transection [24]. Omega-3 fatty acids, including eicosapentaenoic acid (EPA) and docosahexaenoic acids (DHA), have also been suggested to be effective for patients with OA because of their efficacy in attenuating the systemic inflammatory response [25] and cartilage degradation [26].

In this study, we investigated the effect of a dietary supplement BYVET JOINT HEAL^TM^ (BJH, Erebon, Icheon, Republic of Korea), containing green-shell mussel, Collamex^®^ bovine cartilage, shark cartilage, krill extract, and fish oil microencapsulated powders in a rat model with OA induced via injecting monosodium iodoacetate (MIA) on both knees. Subsequently, the efficacy of the supplement was evaluated in dogs with arthritis visiting a veterinary clinic.

## 2. Materials and Methods

### 2.1. Supplement

The commercial supplement BYVET JOINT HEAL^TM^ (BJH, Erebon, Icheon, Republic of Korea) consists of green-shell mussel powder, Collamex^®^ bovine cartilage powder, shark cartilage powder, krill extract powder, and fish oil microencapsulated powder, and is manufactured by Nutrizone Corp. (Auckland, New Zealand). The functional nutrients in this supplement (1000 mg) are listed in Table 1.

### 2.2. Experimental Animals

Six-week-old Sprague-Dawley (SD) rats (Orient Bio Co., Ltd., Seongnam, Republic of Korea) weighing a mean of 150.1 ± 0.1 g were used in the experiment. All experiments were performed in accordance with the animal welfare and use guidelines and after approval was received from the Institutional Animal Care and Use Committee of Eulji University (Approval No. EUIACUC23-24), Republic of Korea. All procedures were conducted in compliance with the guidelines on the principle of regulatory acceptance of 3R (replacement, reduction, and refinements), and all efforts were made to minimize animal suffering. Rats were maintained under standard environmental conditions (a temperature of 23 ± 2 °C, 12 h light–12 h dark cycle, and 55 ± 3% humidity) in a propylene cage (two or three rats per cage) with ad libitum water and food.

A total of 33 male rats were used in the experiments. After a week of acclimatization period, the experimental animals were randomly assigned to four groups: (i) negative control, injected with saline solution in both knee joints of rats and treated with distilled water (DW), orally (n = 5, CON); (ii) positive control, injected with MIA (Sigma Aldrich, St. Louis, MO, USA) in intra-articular space and DW, orally (n = 8, MIA); (iii) BJH treated 689 mg/kg body weight/day, orally administered for 3 weeks starting one day after MIA injection in articular space (n = 10, M+BJH3); and (iv) BJH treated with the same concentration as group (iii) were orally administered before 2 weeks of MIA articular injection and daily supplemented for three weeks (n = 10, BJH2+M+BJH3). The selected dose of 689 mg/kg body weight, according to allometric calculation, corresponds to a nutritive relevant dose of BJH in companion animals (dogs) [27]. DW was used as a vehicle for BJH. Also, MIA rats received DW daily by gastric gavage daily. Rats were dosed once daily with BJH by gastric gavage for 21 days after MIA injection. OA of knee joints was induced on the fourteenth day after initiation of BJH. Initially, rats were anesthetized under isoflurane. Then, both knees of all rats were shaved for injection and disinfected with 70% alcohol. An amount of 3 mg of MIA was dissolved in 50 μL sterile saline and injected into the knee joint cavity through the infrapatellar ligament with a fine needle to induce knee OA. In addition, 50 μL saline was injected into both side knee joints of the control rats. At the end of the BJH feeding period (35th day), rats were weighed and sacrificed under CO_2_.

After sacrifice, the blood of the inferior vena cava and articular cartilage sample of both knee joints were quickly collected. The knee joints of animals were removed from the muscle tissue; the right joints were fixed in 10% neutral buffered formalin, and the left joints were stored at −80 °C for gene expression analysis. The blood samples were collected in EDTA-coated tubes, and the rest were centrifuged (2000× *g* for 10 min); serum samples were collected into a microtube and stored at −80 °C for serum chemistry.

### 2.3. Complete Blood Count and Blood Chemistry

Blood samples were collected three times from the 22nd day from the tail vein into tubes with K3-EDTA and immediately transported to the laboratory. A complete blood count was performed for each whole blood sample within 1 h of the collection by a BC-2800 Vet Auto Hematology Analyzer (MINDRAY, Shenzhen, China) that provides complete blood counts: white blood cells (WBC), lymphocyte (LYM), monocyte (MON), and granulocytes (GRA).

Blood chemistry analysis on serum aliquots was performed by an automatic biochemical analyzer (PT10V, Samsung, Suwon, Republic of Korea) to determine blood urea nitrogen (BUN), creatine (CREA), alkaline phosphatase (ALP), and alanine aminotransferase (ALT).

### 2.4. Analysis of Serum Markers

A microplate reader (Tecan Sunrise^TM^, Tecan, Männedorf, Switzerland) was used to determine serum levels of IL-1β (catalog no: CSB-E08055r, intra CV% < 8% and inter CV% < 10%) and IL-6 (catalog no: CSB-E04640r, intra CV% < 8% and inter CV% < 10%) with rat specific enzyme-linked immunosorbent assay (ELISA) kits according to the manufacturer’s procedure (CUSA BIO, Inc., Houston, TX, USA).

### 2.5. Histopathologic Evaluation

A histological assessment of the femorotibial knee joints of the experimental rats was performed. The right side of each knee joint from the experimental rats was fixed in 10% neutral buffered formalin. Joint cartilage samples blocks were maintained in MoL-DECALCIFIER solution (Total Tissue Diagnostics, Wokingham, UK) using DecalMATE (Milestone Medical, Valbrembo, Italy) to decalcify the inorganic matter in 30 °C for 24 h, dehydrated using an alcohol series and embedded in paraffin blocks. The tissue section was sectioned as 4 μm thick and used for histological assessment with hematoxylin and eosin. An examination of sulfated GAG content within tissue sections was performed after staining with Safranin O/fast green.

### 2.6. Quantitative Real-Time Polymerase Chain Reaction Analysis (QRT-PCR)

Approximately 1 g of the femur tissue containing surface cartilage was disrupted in liquid nitrogen utilizing mortar and pestle, and total RNA was isolated by a Total RNA extraction kit according to the manual. The RNA yield was determined by measuring the absorbance at 260 nm using the Nanodrop. The complementary DNA was synthesized using the easy-spin™ Total RNA Extraction Kit (Intron Biotechnology, Seoul, Republic of Korea). QRT-PCR was performed on StepOnePlus Real-Time PCR System (Applied Biosystems, Waltham, MA, USA, #4376600) 2 × SYBR Green PCR Master Mix (Applied Biosystems, Waltham, MA, USA, #4309155). ß-actin and GAPDH were used as the housekeeping gene, and the fold changes in relative gene expression against ß-actin were calculated using the 2^−ΔΔCt^ method. Sequence-specific primers for cDNA amplified are listed in Table 2.

### 2.7. Clinical Evaluation with Dogs

This clinical evaluation was conducted in several animal hospitals in Korea and included 30 dogs with varying degrees of osteoarthritis signs, no other surgical procedures or metabolic diseases, and who had owners. The breeds and sexes varied, and the ages ranged from 1 to 13 years. The dogs were designed to eat only their regular diet and BJH supplement for 8 weeks. This evaluation was performed in accordance with the animal welfare and use guidelines and after approval was received from the Institutional Animal Care and Use Committee of EreBon Corp. (Approval No. Ere-IACUC 2024-001), Icheon, Republic of Korea. The design of this study evaluated arthritis score indices in dogs before and after BJH administration without a control group, considering ethical issues and the welfare of arthritic dogs.

A joint assessment was performed by a veterinarian using visual scores (an average of individual scores for lameness in walking, trotting, and ramp climbing, ramp descending) and manipulation scores (summation of pain, swelling, crepitus, and mobility reduction) before and 8 weeks after feeding [28]. All parameters were scored on a scale from 0 to 3 (0 = not signs, 1 = mild, 2 = moderate, 3 = severe). The total arthritis score is assessed as the sum of the visual and manipulation scores. Additionally, blood tests were also performed to check the health of the dogs after 8 weeks of feeding. The test methods were the same as in Section 2.3.

The dosage of BHJ supplement is 1 g for dogs weighing less than 5 kg, 2 g for dogs weighing 5 to 10 kg, 3 g for dogs weighing 10 to 20 kg, and 4 g for dogs weighing over 20 kg. It can be fed alone or mixed with food/snacks. During the feeding period, the owner should check for any abnormalities such as vomiting, diarrhea, decreased appetite, decreased energy, or itchiness. If any abnormalities occur, the owner should immediately stop feeding and contact the responsible veterinarian.

### 2.8. Statistical Analysis

Data of body weight, blood count, blood biochemistry, cytokine, relative gene expression, and clinical data, including visual score, manipulation score, and total arthritic score, are expressed as mean ± standard error of the mean (SEM). Body weight change and hematological changes were assessed through a two-way repeated measures ANOVA followed by Bonferroni post hoc tests when necessary. Statistical data of serum chemistry, cytokine, and gene expressions were analyzed using one-way analysis of variance (ANOVA) with Dunn’s multiple comparisons using GraphPad Prism 3 software (version 3.0, San Diego, CA, USA). Non-normally contributed data were analyzed using the Kruskal–Wallis test followed by Dunn’s multiple comparison tests. Also, Bonferroni corrections were applied. Clinical evaluation was analyzed by a paired *t*-test with Wilcoxon-matched paired posttests. A *p*-value less than or equal to 0.05 is judged as statistically significant.

## 3. Results

### 3.1. Body Weight Assessment

**Figure 1:**
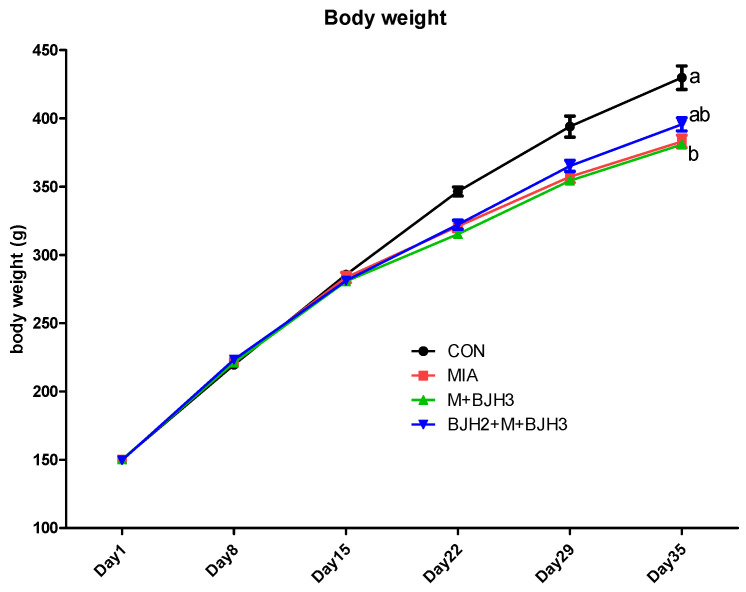
Comparison of mean body weights throughout the five-week experimental period: (i) CON—negative control; (ii) MIA—positive control, injected with monosodium iodoacetate (MIA) on day 14; (iii) M+BJH(BYVET JOINT HEAL^TM^)3—orally administered for three weeks starting a day after MIA injection; (iv) BJH2+M+BJH3—BJH orally administered two weeks before MIA articular injection and supplemented daily for three weeks. Labeling on the same day with a different letter indicates significance at *p* < 0.05.

The mean body weights of the rats in each experimental group were determined over five weeks (Figure 1). The body weight values obtained for the CON group are within the standard values for the corresponding age of SD rats. After MIA articular injection, CON had higher mean body weights than the other groups. However, there were no significant differences. The preventive treatment group (BJH2+M+BJH3) showed similar body weights compared to those of the control on Day 35. The preventive treatment group (BJH2+M+BJH3) showed apparent preventive effects of treatment on body weight.

### 3.2. Hematology and Blood Chemistry

**Figure 2:**
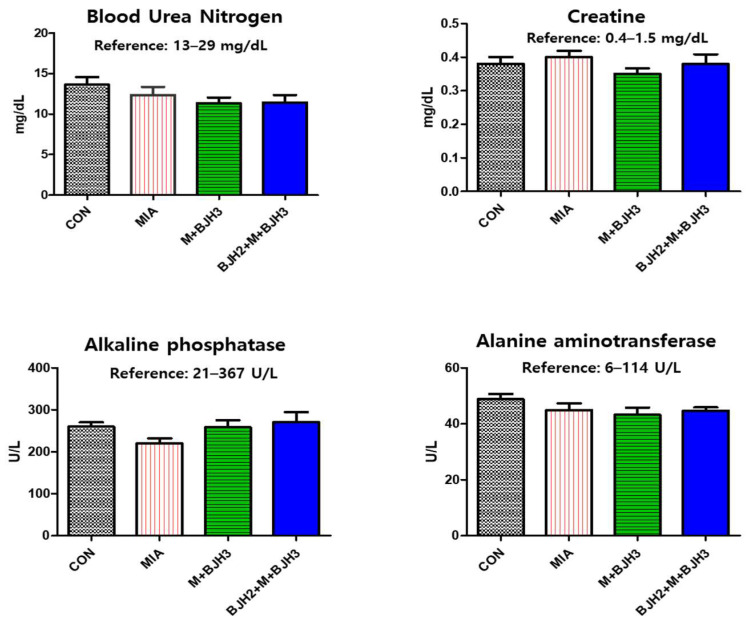
Serum chemistry parameter analysis results of the rat models. All parameters of all groups are within normal references.

Serum chemistry parameters are used as biomarkers for diagnosing organ or tissue injuries, such as impaired kidney and liver function, in clinical practice. All parameters of blood chemistry, including blood urea nitrogen, creatine, alkaline phosphatase, and alanine aminotransferase, fell within the normal range of SD rats (Figure 2), and no signs of adverse effects were observed after the clinical examination. Hence, supplementation was well-tolerated and had no harmful effect on kidney and liver functions even after oral administration for up to five weeks.

**Figure 3:**
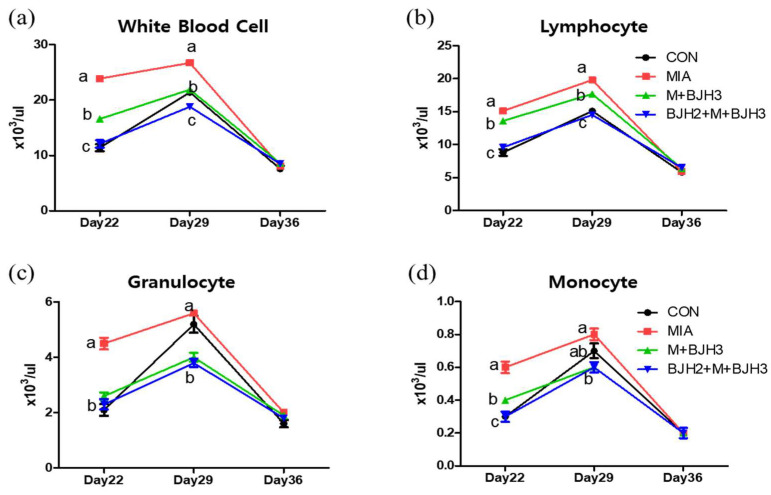
Hematological changes: (**a**) white blood cells, (**b**) lymphocyte, (**c**) granulocyte, and (**d**) monocyte 8 (day 22), 15 (day 29), and 22 (day 36) days after monosodium iodoacetate (MIA) injection. Labeling on the same day with a different letter indicates significance at *p* < 0.05.

In the hematology analysis (Figure 3), white blood cells (WBCs) of MIA were significantly higher compared with CON and the other two experimental groups on days 22 and 29. However, no differences were observed between the groups on the final examination day (day 36). Lymphocyte results were also like those of WBCs. The differential granulocyte results showed that the MIA had the highest number of granulocytes on day 22 day among all groups, had similar numbers compared to CON, and had a higher number than the two treated experimental groups (M+BJH3 and BJH2+M+BJH3) on day 29. Eight days after MIA injection (day 22), monocytes of MIA were also highest among all groups, and the number of monocytes in M+BJH3 was higher than that in CON and BJH2+M+BJH3.

### 3.3. Serum IL-6 and IL-1β of Rats

**Figure 4:**
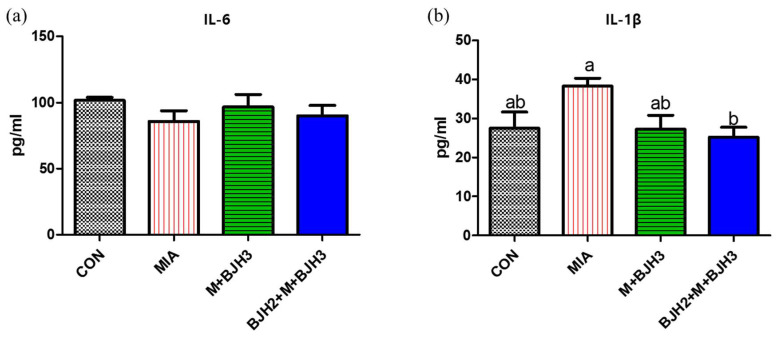
Serum interleukin (IL)-6 and IL-1β levels on the day 36 of the experiment: (**a**) IL-6 and (**b**) IL-1β levels. A different letter indicates a statistically significant difference (*p* < 0.05).

Figure 4 illustrates that BJH treatment showed no effect on serum IL-6 levels. However, OA-induced rats without BJH treatment (MIA) showed a significant increase in serum IL-1β compared to that in the BJH2+M+BJH3 group.

### 3.4. Relative Gene Expression of OA and Inflammation Factors

**Figure 5:**
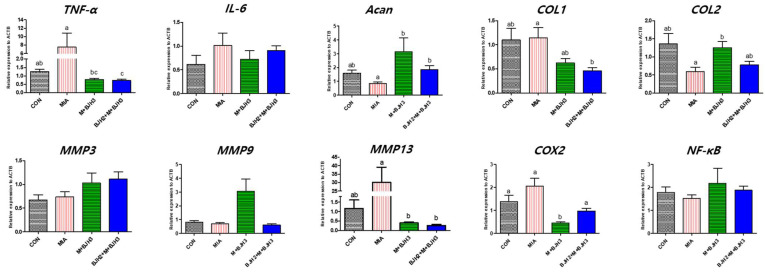
Relative quantification of mRNA expression levels of ten genes in the articular tissue of each group, which has been normalized with the reference gene (*ACTB*) expression using quantitative real-time polymerase chain reaction. The graph shows the data of each gene evaluated for each in six replicates and three independent experiments. A different letter indicates a statistically significant difference (*p* < 0.05).

The relative gene expression of articular cartilage and bone tissue of knee joints is shown in Figure 5. *TNF-α* expression was highest in MIA. *TNF-α* expression in CON and M+BJH was similar; however, that in BJH2+M+BJH3 was significantly the lowest (*p* < 0.05). The *aggrecan (Acan)* expression level was significantly lower than that of MIA in the four groups and was similar in both groups that started BJH treatment before and after MIA injection (*p* < 0.05). The *collagen type I* (*COL1*) expression level in MIA was similar to that in CON, whereas it was lowest in BJH2+M+BJH3 (*p* < 0.05). The lowest *collagen type II (COL2)* expression was found in MIA, whereas it was highest in M+BJH3 (*p* < 0.05). The *MMP13* expression level was highest in MIA and was significantly lower in M+BJH3 and BJH2+M+BJH3 compared with that in MIA (*p* < 0.05). *Cyclooxygenase-2 (COX2)* expression was significantly lower in M+BJH3 compared with that in the other three groups. Genes that did not show significant differences in expression between groups included *IL-6*, *MMP3*, *MMP9*, and *nuclear factor-κβ (NF-κβ)*.

### 3.5. Effect of BJH Observed in the Histological Assessment

**Figure 6:**
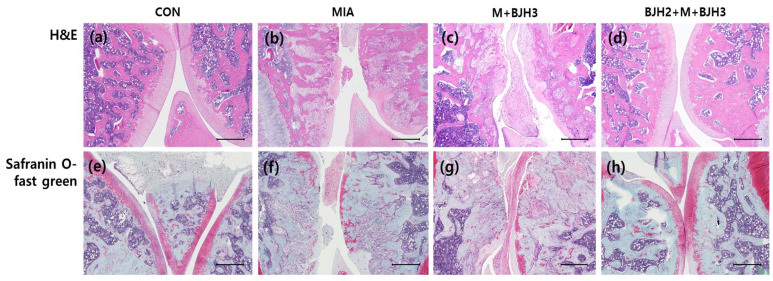
Hematoxylin and eosin (H&E) and safranin O/fast green staining of rat knee joints. H&E staining showing the following: (**a**) CON—Normal histological findings with smooth cartilage surface in negative control. (**b**) MIA—Severe lesions, weak stinging of chondrocyte nuclei, and fibrillation with irregular cartilage surface in positive control. (**c**) M+BJH3—Irregularity in articular surface and inflammatory cells. (**d**) BJH2+M+BJH3—Intact and regular cartilage surface with normal chondrocytes. Safranin O/fast green staining showing the following: (**e**) Normal cartilage and intact surface in CON. (**f**) Loss of safranin O/fast green positive staining and cartilage loss in MIA. (**g**) Few cell clusters and more positive safranin O/fast green staining observed in M+BJH3. (**h**) Intact cartilage surface and similar finding as (**e**). Forty-times magnification.

After safranin O/fast green staining, a histological examination revealed GAG loss in the experimental section of the knee in the MIA. Severe loss of safranin O/fast green staining was observed, showing cartilage damage in the femorotibial joints. The cartilage surface of the femorotibial joints of the BJH2+M+BJH3 group was similar to those of CON, which indicated a normal appearance of the articular cartilage (Figure 6a,e). Overall, these results demonstrate that BJH can have promising effects in preventing articular cartilage damage-related diseases.

### 3.6. Effect of BJH Observed in Clinical Assessment

All 30 dogs were clinically confirmed arthritic adult dogs. A broad range of dog sizes was represented: 1 medium (11–25 kg) and 29 small breeds (<11 kg), encompassing 11 different dog breeds. All dogs were fed BJH supplements for 8 weeks without any problems (vomiting, diarrhea, anorexia, food allergy, etc.). The owners reported good palatability and ease of administration of the BJH supplement. The veterinarians performed physical examinations, blood tests, and joint evaluations. All dogs had no specific abnormalities found in the physical examinations and blood tests. After 8 weeks of feeding, 17 of the 30 dogs (56.67%) had a decrease in visual score, 23 (76.67%) had a decrease in manipulation score, and 24 (80.00%) had a decrease in total arthritis score. The veterinarians concluded that the BJH supplement was effective (Table 3).

**Figure 7:**
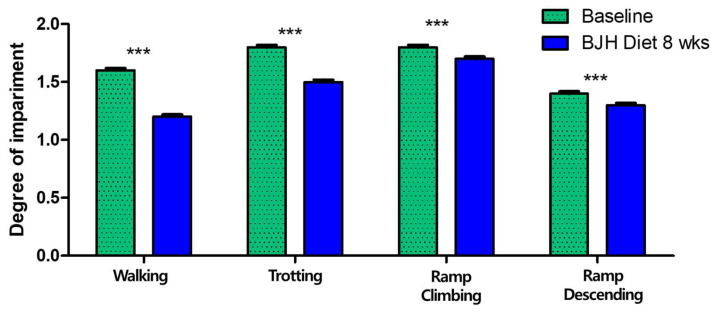
Changes in walking, trotting, ramp climbing, and ramp descending between D0 (Baseline) and 8 weeks of BJH diet supplement (BJH Diet 8 weeks). The values are means ± SEM, n = 30 dogs, *** *p* < 0.05, paired *t*-test with Wilcoxon matched paired posttests.

After 8 weeks of feeding, the mean values of the visual scores significantly changed from 1.57 to 1.17 for walking, from 1.80 to 1.50 for trotting, from 1.77 to 1.73 for ramp climbing, and from 1.37 to 1.33 for ramp descending (*p* < 0.05) (Figure 7). Twelve of the thirty dogs (40.00%) had a decrease in walking score, nine (30.00%) had a decrease in trotting score, nine (30.00%) had a decrease in ramp climbing score, and one (3.33%) had a decrease in ramp descending score.

**Figure 8:**
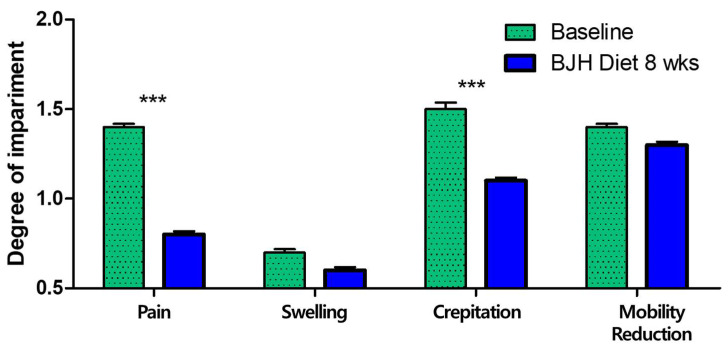
Changes in each manipulation index (pain, swelling, crepitus, and mobility reduction) between D0 (Baseline) and 8 weeks of BJH diet supplement (BJH Diet 8 weeks). The values are means ± SEM, n = 30 dogs, *** *p* < 0.05, paired *t*-test with Wilcoxon matched paired posttests.

After 8 weeks of feeding, the mean values of the manipulation scores significantly changed from 1.37 to 0.80 for pain, from 0.67 to 0.60 for swelling, from 1.47 to 1.07 for crepitation, and from 1.37 to 1.30 for mobility reduction. Among these, significant changes were observed for pain and crepitation (*p* < 0.05) (Figure 8). Seventeen of the thirty dogs (56.67%) had a decrease in pain score, two (6.67%) had a decrease in swelling score, thirteen (43.33%) had a decrease in crepitation score, two (6.67%) had a decrease in mobility reduction score.

**Figure 9:**
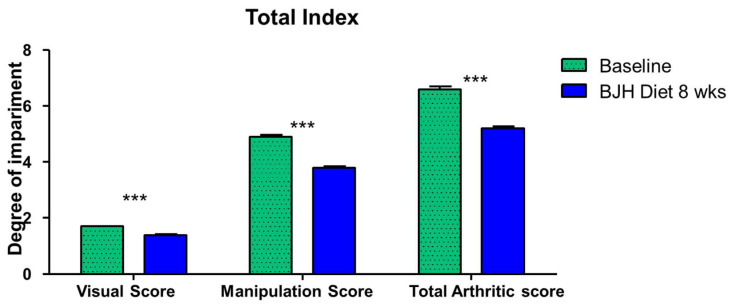
Changes in the “visual score”, the “manipulation score”, and the “total arthritic score” between D0 (Baseline) and 8 weeks of BJH diet supplement (BJH Diet 8 weeks). The values are means ± SEM, n = 30 dogs, *** *p* < 0.05, paired *t*-test with Wilcoxon matched paired posttests.

Overall, the visual score significantly changed from 1.63 to 1.14, the palpation score from 4.80 to 3.77, and the total joint score from 6.43 to 5.20 (*p* < 0.05) (Figure 9). The ‘Visual Score’, ‘Manipulation Score’, and ‘Total Arthritic Score’ decreased by an average of 11.79%, 21.53%, and 19.07%, respectively.

## 4. Discussion

In this study, we demonstrated that a new dietary supplement developed in Erebon (BYVET JOINT HEAL^TM^, Icheon, Republic of Korea) prevented cartilage deterioration and improved serum inflammatory parameters and cartilage repair-related signaling pathways in MIA-induced OA rats without causing any harmful effects on liver and kidney functions.

The body weight change in all groups was similar to normal weight gain until day 15, a day after OA was induced. SD rats are an outbred strain characterized by a spontaneous increase in body weight when fed standard laboratory chow ad libitum. However, from day 22, a significant difference was observed between the CON and the other three groups (MIA, M+BJH3, and BJH2+M+BJH3), showing that OA caused a change in body weight. In particular, the body weight results on day 35, the last day of BJH administration, were lower in the preventive treatment group (BJH2+M+BJH3) than CON but significantly higher in MIA and M+BJH3 groups. This indicates that administering this dietary supplement normally promotes their body weight development, even if OA is subsequently induced or occurs.

All results of hematology analysis were similar on day 36, with no significant differences between groups, which indicated that three weeks after OA induction, inflammatory immune cells in the systemic blood appeared similar regardless of OA occurrence, and OA appeared to be localized in both knees. The preventive treatment group (BJH2+M+BJH3) showed a similar number of WBCs and LYM as those of CON. The result that the WBC and LYM of the peripheral blood in the preventive treatment group remained similar to that of CON indicates that preventive treatment of BJH can continuously maintain peripheral blood without changes, similar to a normal individual without pathologic changes.

In OA formation, cartilage, synovial inflammation, and many inflammatory cytokines are known to be involved. A previous study showed that serum TNF-α and IL-6 levels increased after MIA injection, which decreased with the type II collagen supplementation. IL-1β levels showed no difference after type II collagen oral administration [29]. Likewise, in this study, the IL-1β level was significantly lower in the preventive treatment group (BJH2+M+BJH3) compared with the MIA; however, no significant difference was observed in the group treated with BJH after OA induction (M+BJH3) compared with the MIA. This is probably because type II collagen treatment in the previous study was performed only seven days before MIA induction; however, our preventive care was performed 14 days before OA induction. IL-1β is a proinflammatory cytokine that is most abundant and plays a central role in OA pathogenesis and inflammatory response [30]. When IL-1β binds to IL-1RI, it can activate a cascade of signaling pathways that causes OA.

Mitogen-activated protein kinase (MAPK) signaling has been demonstrated to be a predominant catabolic pathway of cartilage damage. MAPK activates a kinase subfamily that decreases aggrecan and type II collagen. When MAPK signaling is activated, p38 MAPK and c-Jun N terminal kinases are activated to suppress aggrecan and type II collagen expression, which ultimately decreases chondrocyte synthesis [31]. Similar results were confirmed in our study. Considering the expression levels of *aggrecan* (*ACAN*) and *type II collagen* (*COL2*), BJH oral administration can be assumed to have downregulated MAPK signaling in the OA rat model, thereby operating a mechanism that repairs cartilage damage. The p38 MAPK and c-Jun N terminal kinases activate MMPs and ADAMTS, which stimulate aggrecanases and collagenases to reduce extracellular matrix synthesis and proteoglycan production [32].

TNF-α, a member of the TNF family, is a proinflammatory cytokine that is a catabolic factor of cartilage homeostasis [33,34,35]. Not only soluble TNF- α, but also its precursor form, transmembrane TNF- α, is involved in the inflammatory response. Both forms function through two receptors, each performing a particular function: activating TNF receptor 1 (TNFR1) and TNF receptor 2 (TNFR2). The proinflammatory effect of TNF-α may rely on TNFR1. The binding of these TNF-α and TNFR1-related proteins stimulates NF-κB, proinflammatory cytokines, MAPK and its subfamilies, and activated protein-1, which disrupts collagen and degrades proteoglycan [36]. Both IL-1β and TNF-α are known to be synthesized by the synovium and potentiate MMP2 upregulation, which participates in macrophage-mediated joint damage in dogs [37]. Therefore, oral administration of BJH to companion animals such as dogs can be expected to prevent OA via significantly reducing both *IL-1β* and *TNF-α* levels derived from joint cartilage damage, as demonstrated in this study.

Both IL-1β and TNF-α can inhibit chondrogenesis via the NF-κB pathway [5] and promote MMP release from the synovial fibroblasts in cartilage destruction [33,34]. In particular, among various MMPs in OA, MMP1, MMP3, and MMP13 have been reported to have increased expression in OA and are closely related to OA development. This could explain the lower expression level of *MMP13* in the BJH-treated groups (M+BJH3, BJH2+M+BJH3) compared with that in the positive control (MIA). Oral administration of BJH can be suggested to inhibit *TNF-α*, a powerful proinflammatory cytokine around joints; this can reduce MMP13 expression. IL-1β increased MMP expression in chondrocytes [38], and this is known to be a primary mechanism of OA [39]. MMPs not only destroy cartilage matrix components but also inhibit their synthesis. In chondrocytes, MMP13 inhibited the synthesis of type II collagen and aggrecan, the main components of the cartilage. When chondrocytes were stimulated with IL-1β, MMP expression increased, whereas that of type II collagen and aggrecan decreased [38]. Likewise, our study results showed that the expression of *ACAN* and *COL2* in the BJH-treated groups (M+BJH3, BJH2+M+BJH3) was higher than that in MIA; *MMP13* expression in MIA was higher than that in the BJH-treated groups. This is the result of stimulating cartilage cells via IL-1β in the area where OA occurred in MIA without BJH treatment.

IL-6 is also known to contribute to the OA inflammatory response. It has been shown to participate in the anti-inflammatory response and regenerative process [40,41]. In patients with high-graded OA, significantly greater IL-6 concentrations are detected in the synovial fluid, which is hypothesized to have a predictive biomarker of OA progression [42]. TNF-α and IL-1β can activate the NF-kB pathway that provokes proinflammatory cytokines such as IL-6 while inducing cyclooxygenase-2 and MMP production [43]. In our study, all groups showed no differences in serum IL-6 and articular cartilage bone relative gene *IL-6* expression levels.

After confirming the results of the rat experiment, a clinical efficacy evaluation of the BJH supplement was conducted on 30 dogs diagnosed with joint abnormalities from several animal hospitals. After feeding the BJH supplement for 8 weeks, the veterinarian determined that the supplement was effective in reducing arthritic signs, and no other adverse effects were observed in the clinical examination. Long-term treatment with medications is an option for dogs with arthritis, but this can increase the risk of side effects in most patients. An alternative approach is to use natural ingredients to treat OA in both humans and animals [15,21,22].

Low-molecular-weight polysulfate glycosaminoglycans (PSGAGs) stimulate the synthesis of pure collagen and GAGs in cartilage tissue. Arthritic tissues are more sensitive to PSGAG stimulation. These results provide direct evidence for the true chondroprotective role of chondroitin-rich low-molecular-weight PSGAGs in the treatment of degenerative joint diseases [24,44]. Chondroitin sulfate has been shown to impede the progression of structural changes in joint tissues and is used in the management of patients with osteoarthritis [24,45,46]. Therefore, it is thought that the low-molecular-weight GAGs and chondroitin components of BJH may help in the regeneration of articular cartilage in dogs.

Feeding arthritic dogs a green-lipped mussel-supplemented diet resulted in an overall positive impact on their arthritic signs (visual score, manipulation score, total arthritic score) [47,48,49]. In many studies, marine-based fatty acid compounds, consisting of a proprietary extract of stabilized marine lipids from the New Zealand green-lipped mussel (*Perna canaliculus*), have been reported to be associated with beneficial outcomes in clinical OA cases in dogs [50,51,52,53]. The anti-inflammatory properties of green-lipped mussel powder are attributed to its various pharmacologically active components. The most commonly known components are ETA (eicosatetraenoic acid), EPA, and DHA. ETA is found only in green-lipped mussels and binds cyclooxygenase, which is an enzyme that causes inflammation. Our study also confirmed that the anti-inflammatory action of fatty acid compounds contained in green-lipped mussel powder, krill extract, and fish oil powder helped improve arthritis symptoms.

When 30 dogs were fed 40 mg of undenatured type II collagen (UC-II) per day for 30 days, it was able to improve mobility in dogs with OA [54]. According to Gupta et al., active UC-II supplementation alone (10 mg/day for 150 days) was well tolerated and significantly improved well-being in dogs with moderate arthritis [55]. Similarly, our results showed that when 30 dogs were fed 48 mg of UC-II per day for 8 weeks, it improved mobility in dogs with OA. Studies reported so far have shown that it is difficult to obtain sufficient effects with only one type of functional ingredient in dogs with OA, so many studies have been conducted to evaluate the efficacy of nutritional supplements that combine multiple types of functional ingredients [56,57,58]. Therefore, as shown in the results of this study, long-term intake of nutritional supplements that combine functional ingredients that help regenerate cartilage and relieve inflammation in joints is thought to be helpful in alleviating arthritic signs in dogs. For future research, it is necessary to consider the age, sex, and breed diversity of companion dogs to distinguish the stages of OA and to have a larger sample. In addition, in order to see the preventive effect, it will be necessary to administer BHJ before the onset of OA and evaluate it in breeds with a high risk of OA or obese individuals.

## 5. Conclusions

In conclusion, we report that BJH, a dietary supplement containing mucopolysaccharide protein, chondroitin sulfate, type II collagen, and omega-3 fatty acids, effectively reduces cartilage damage via promoting the aggrecan and type II collagen synthesis, inhibiting MMP13 synthesis and prevents OA-induced pathologic degeneration via diminishing inflammatory factors such as TNF-α and IL-1β. Particularly, no adverse effects were observed. Also, the veterinarian determined that the supplement was effective in reducing arthritic signs, and no other adverse effects were observed in clinical trials with dogs. Therefore, we successfully demonstrated that this dietary supplement could be beneficial in alleviating cartilage damage and degeneration and preventing OA.

## Figures and Tables

**Table 1:** Composition of supplement of BYVET JOINT HEAL^TM^.

Nutrients	Contents (mg/g) in Supplement (mg/g)
Mucopolysaccharide protein	600
Chondroitin sulfate	120
Type II collagen	48
Omega 3 fatty acid	50

**Table 2:** Primer set sequence used for real-time PCR.

*Gene*	*Official Full Name*	*Primer Sequence*	*Gene Bank No.*
*TNF-α*	tumor necrosis factor-alpha	ACAAGGCTGCCCCGACTAT	X66539.1
		CTCCTGGTATGAAGTGGCAAATC	
*IL-6*	interleukin 6	GCCCTTCAGGAACAGCTATGA	M26744.1
		TGTCAACAACATCAGTCCCAAGA	
*Acan*	aggrecan	GAAGTGGCGTCCAAACCAA	NM_022190.1
		CGTTCCATTCACCCCTCTCA	
*COL1*	collagen type I	CTGGTGAACAGGGTGTTCCT	BC133728
		GGAAACCTCTCTCGCCTCTT	
*COL2*	collagen type II	CGAGGTGACAAAGGAGAAGC	L48440
		AGGGCCAGAAGTACCCTGAT	
*MMP3*	matrix metallo proteinases 3	GAGTGTGGATTCTGCCATTGAG	NM_133523.2
		TTATGTCAGCCTCTCCTTCAG A	
*MMP9*	matrix metallo proteinases 9	GCGCCAGCCGACTTATGT	NM_031055.1
		AATCCTCTGCCAGCTGTGTGT	
*MMP13*	matrix metallo proteinases 13	ACGTTCAAGGAATCCAGTCTC	NM_133530.1
		GGATAGGGCTGGGTCACACTT	
*COX2*	cyclooxygenase isoform COX-2	AGAGAAAGAAATGGCTGCAGAGT	S67722.1
		AGCAGGGCGGGATACAGT	
*NF-kB*	nuclear factor kappa B subunit 1	GCACCAAGACCGAAGCAATT	NM_001276711.1
		GAAACCCCACATCCTCTTCCTT	
*ACTB*	β-actin	AGGCCAACCGTGAAAAGATG	NM_031144.3
		ACCAGAGGCATACAGGGACAA	

**Table 3:** Results of veterinary joint evaluation before and after 8 weeks of taking BJH supplement, including signalment on 30 dogs.

No.	Breed	Age (Years)	Gender	Body Weight (kg)		Joint Evaluation					
						Visual Score		Manipulation Score		Total Arthritic Score	
				Before	8 Weeks	Before	8 Weeks	Before	8 Weeks	Before	8 Weeks
1	Maltese	6	CM	2.98	3.07	1.00	1.00	3.00	2.00	4.00	3.00
2	Cocker Spaniel	10	FS	10.90	11.2	1.50	1.00	4.00	3.00	5.50	4.00
3	Poodle	10	FS	2.70	2.70	2.00	2.00	5.00	3.00	7.00	5.00
4	Poodle	10	FS	4.80	4.90	1.00	1.00	1.00	2.00	2.00	3.00
5	Chihuahua	7	CM	3.25	3.47	2.00	1.50	6.00	5.00	8.00	6.50
6	Poodle	7	FS	4.34	4.38	1.00	1.00	1.00	1.00	2.00	2.00
7	Bichon Frise	1	FS	4.87	4.90	1.75	1.75	5.00	4.00	6.75	5.75
8	Bichon Frise	4	FS	2.90	3.10	2.00	2.00	6.00	5.00	8.00	7.00
9	Maltipoo	4	CM	4.00	4.10	1.00	1.00	1.00	0.00	2.00	1.00
10	Poodle	4	CM	2.70	2.70	1.50	1.50	1.00	2.00	2.50	3.50
11	Japanese Spitz	4	CM	6.80	6.88	1.50	2.00	5.00	4.00	6.50	6.00
12	Maltese	4	CM	5.98	5.91	2.25	2.00	8.00	6.00	10.25	8.00
13	Japanese Spitz	10	CM	4.90	5.10	2.00	2.00	6.00	6.00	8.00	8.00
14	Maltese	11	CM	5.20	5.26	2.25	2.00	7.00	5.00	9.25	7.00
15	Pomeranian	1	CM	2.34	2.47	2.00	2.00	5.00	4.00	7.00	6.00
16	Poodle	9	CM	4.80	4.68	1.25	1.00	6.00	3.00	7.25	4.00
17	Japanese Spitz	9	CM	10.20	10.50	1.75	1.50	5.00	4.00	6.75	5.50
18	Pomeranian	6	CM	3.90	3.80	0.75	0.75	2.00	2.00	2.75	2.75
19	Pekingese	4	CM	6.60	6.72	1.25	1.00	3.00	3.00	4.25	4.00
20	Maltese	7	FS	3.08	3.12	1.75	1.00	6.00	4.00	7.75	5.00
21	Maltese	7	FS	3.46	3.40	2.25	1.75	8.00	6.00	10.25	7.75
22	Maltese	9	FS	2.46	2.20	2.00	1.50	6.00	4.00	8.00	5.50
23	Bichon Frise	4	FS	6.86	6.90	1.50	1.25	4.00	3.00	5.5.	4.25
24	Mixed	13	FS	3.90	4.00	1.75	1.50	9.00	7.00	10.75	8.50
25	Poodle	4	CM	3.48	3.52	0.50	0.50	1.00	1.00	1.50	1.50
26	Poodle	7	CM	6.30	6.20	1.50	1.25	5.00	4.00	6.50	5.25
27	Maltipoo	2	CM	2.00	1.90	1.75	1.50	7.00	6.00	8.75	7.50
28	Shiba Inu	6	CM	11.60	12.00	1.50	1.00	3.00	2.00	4.50	3.00
29	Pomeranian	1	FS	2.50	2.60	2.00	1.75	7.00	5.00	9.00	6.75
30	Maltese	7	CM	3.46	3.60	2.50	2.00	8.00	7.00	10.50	9.00

CM, castrated male; FS, female spayed.

## Data Availability

All relevant data are within the manuscript.
